# First-Time Mothers Have a Desire to Be Offered Professional Breastfeeding Support by Pediatric Nurses: An Evaluation of the Mother-Perceived-Professional Support Scale

**DOI:** 10.1155/2019/8731705

**Published:** 2019-08-06

**Authors:** Matilda Möller Ranch, Sofia Jämtén, Stina Thorstensson, Anette C. Ekström-Bergström

**Affiliations:** ^1^Neonatal Care Unit, NÄL Hospital Trollhättan, Sweden; ^2^Pediatric Healthcare Setting, Capio, Lysekil, Sweden; ^3^School of Health Sciences, University of Skövde, Skövde, Sweden; ^4^Department of Health Sciences, University West, Sweden

## Abstract

**Background:**

Although the World Health Organization recommends exclusive breastfeeding for six months, the rate of breastfeeding has decreased worldwide. Breastfeeding is the natural way of feeding a baby, but it is a process that has to be learnt. It is not unusual for problems to occur and hence support for breastfeeding is vital. The aim of this study was to explore first-time mothers' experiences of the breastfeeding support offered by pediatric nurses, as well as to develop and evaluate the Mother Perceived Support from Professionals (MoPPS) scale.

**Methods:**

A qualitative design involving both inductive and deductive approaches was chosen. Nine first-time mothers were interviewed regarding their experiences of the breastfeeding support offered by pediatric nurses. Semistructured interviews were conducted. The mothers were also asked to grade their experiences of breastfeeding support on the MoPPS scale. A qualitative content analysis was applied when analyzing the data obtained using both the inductive (interviews) and deductive (MoPPS scale) approaches.

**Results:**

The results revealed that the mothers felt the desire to breastfeed, although they all experienced some difficulties. They wanted the pediatric nurses to be perceptive and provide professional support based on their own experiences. When the pediatric nurses took time and booked extra appointments, the mothers felt supported. The inductive analysis resulted in one theme: “When wanting to breastfeed, mothers have a desire to be offered professional breastfeeding support”. Two main categories were identified, namely “Mothers wanted but lacked breastfeeding support” and “Mothers received professional support.” The deductive analysis of the MoPPS scale showed similar results, and the questions were perceived as relevant to the aim. The mothers considered it important that the pediatric nurses had sufficient knowledge about breastfeeding. It was also considered important that the pediatric nurses involved the mothers' partners in the breastfeeding support. Therefore, we suggest that these areas should be included in the MoPPS scale for pediatric nurses.

**Conclusions:**

The MoPPS scale can be a useful tool for helping pediatric nurses to offer mothers professional breastfeeding support. Indeed, when offering breastfeeding support, pediatric nurses can use the items included on the MoPPS scale as guidance.

## 1. Background

The amount of exclusively breastfed babies varies significantly among countries, although globally approximately 40% of babies under the age of six months are exclusively breastfed [1]. In Sweden, an ongoing reduction in the rate of breastfeeding of babies under four months of age was observed between 2004 and 2015. Further, statistics from 2015 show that 51.2% of all four-month-old babies in Sweden were exclusively breastfed, while 23% were partially breastfed [2]. Indeed, the breastfeeding rates have decreased over time among all social groups in Sweden [3].

Although breastfeeding is a natural process, it is still a process that has to be learnt [4]. Research shows that most women experience at least one problem with their breasts during breastfeeding, for example, sore nipples, milk jams, low milk production, or difficulties with latching which might explain why mothers ceased breastfeeding [5]. Other research shows similar results; the main reasons why mothers quit breastfeeding have been identified as poor breastfeeding support (such as offering formula for nonmedical reasons), lack of knowledge about breastfeeding, low self-efficacy, problems with latching, and belief that they have low milk production [6]. A new mother has to learn both how to meet her baby's needs and how to apply various lactation techniques [7].

In order to improve maternity care as well as to protect, promote, and support breastfeeding, the World Health Organization (WHO), together with the United Nations Children's Fund (UNICEF), launched the Baby-Friendly Hospital Initiative (BFHI) in 1991 [4]. One of the global goals of the initiative was to allow all women to exclusively breastfeed their baby for the first six months [8]. The BFHI describe ten steps that are intended as guidelines that all health-care staff who work with children should follow [8], including the requirement to help mothers start and maintain lactation and then breastfeed on demand [9, 10]. Pediatric health-care settings are considered important and necessary if mothers are to continue breastfeeding after childbirth [6]. Pediatric nurses can identify and help mothers to overcome breastfeeding difficulties early on, and they can provide new mothers with evidence-based information and preventive guidance [11].

The nurses employed in pediatric health-care settings in Sweden follow children's development from birth until the age of five years, meeting the family within the first week after being discharged from the maternity ward [12]. They can hence support breastfeeding-friendly practices through the regular meetings that are offered in such settings [11, 12]. For example, nurses could inform mothers about the benefits of breastfeeding, how to get started, how to prevent problems, and how to maintain lactation. It is important that nurses strive to strengthen and support each individual parent in the situation he or she finds him/herself in [6, 12]. A multifaceted cooperative approach is critical to the success of the process of maintaining breastfeeding. In this regard, three of the most effective interventions are providing early professional breastfeeding support, increasing the self-esteem of mothers and educating both mothers and staff about breastfeeding [6].

Previous studies have shown support to be of key importance if mothers are to continue breastfeeding. Mothers have reported a preference for different kinds of support, including social support from family, parent groups, and social media, as well as professional support [13]. In this context, the notion of support can thus be divided into two different types, namely, social support and professional support. Social support is provided by those we have a relationship with, such as a partner or mother, and hence it is built on trust. Professional support requires the patient to trust a professional from the first meeting onwards, although the professional does not have to trust the patient. This gives rise to an asymmetric relationship built on the specific reason for the meeting [14]. A new mother has access to professional support through various health-care professionals, including midwives, nurses, and other health-care staff who are around her when the baby is newborn [13]. In a study from Sweden the results show that professional support is perceived to be positive when health-care professionals listen to and accommodate an individual woman's needs. They also demonstrated that the credibility of professional support is strengthened when the involved professionals create an open-minded environment that allows the mother to speak freely based on her own experiences [15], that is, an environment in which the nurse can see and accommodate the individual patient and nursing is performed in partnership with that patient [16]. In another study from Sweden the mothers express a need to be reassured regarding their breastfeeding situation. They reported feeling safer regarding breastfeeding if health-care staff were present during lactation and reassured them as to their technique. It is considered equally important to obtain advice about how best to read the child's signals, test different breastfeeding positions, and handle any problems that arose. The mothers also want to be dealt with based on their own unique situation and needs [17].

When seeking to develop and evaluate care routines and care interventions, it is important to use validated scales. The Mother Perceived Support from Professionals (MoPPS) scale was developed using theory about informational, instrumental, and emotional support [18] and it is intended to explore mothers' experiences of professional support [19]. The MoPPS scale is currently being validated in several studies [20, 21]. Such professional support is seen as an important aspect in relation to helping mothers to maintain breastfeeding [13] and no adequate scale to assess mothers' perception of professional support is available. Pediatric nurses working in child health-care centers represent the principal group of professionals who are in contact with the family when a baby is newborn [12]. The aim of the present study was therefore to explore first-time mothers' personal experiences of the breastfeeding support provided by pediatric nurses, as well as to develop and evaluate the MoPPS scale.

## 2. Methods

### 2.1. Design

A qualitative design involving both inductive and deductive approaches was chosen to address the aim of this study. Open questions accounted for the inductive part of the study, meaning that empirical data were collected unconditionally. A deductive approach involves choosing an instrument or theory and then using it empirically [22]. In the present study, the chosen instrument was the MoPPS scale. Semistructured interviews were used to explore mothers' experiences and allow them to speak freely about the professional support they had received. By following such an approach, the focus can be directed in a unique way toward the phenomenon to be investigated [23]. The researchers thus used an interview guide, which, according to Polit and Beck, is necessary so that nothing is overlooked [24]. A qualitative content analysis was used when analyzing the data [22]. This method is useful when the aim is to examine and interpret text such as interview transcripts, with the focus being on identifying similarities and differences in the material [22].

### 2.2. Participants and Setting

The inclusion criteria for the study were that the participants should be first-time mothers, aged at least 19 years old, and able to speak Swedish. The child should be at least three months and not older than five months, and he/she should not have received care in a neonatal care unit after birth. The mother should have breastfed the baby at some point after birth, although it was not necessary to still be breastfeeding at the time of the study. It was not necessary for the mothers to have experienced any breastfeeding problems to be included in the study.

A request for participants in the study was posted on social media (Facebook) in different discussion groups, with the aim of obtaining a wide geographical spread and include participants from urban, suburban, rural and exurban settlements. The participants were identified by means of convenience sampling, which means that they made contact with the researcher themselves [23]. In total, nine women were interviewed during the study. They had an age range of 23–39 years old. All the participants lived with a partner. Two of the participants were acquainted to one of the researchers. None worked as pediatric nurses. Their educational level varied from high school to university, and they resided in small (population: 19600) to large cities (population: 2,2 millions). Further, all the participants were first-time mothers, and they all spoke Swedish. The age range of the babies was 3–4(1/2) months old when the interviews were conducted.

### 2.3. Data Collection

The participants contacted the researchers by email after they had seen the request to participate in the study on social media. All of them received an information letter, either by email or handed out at a meeting. Three interviews were conducted in person, while six were conducted by telephone. The interviews were recorded and transcribed verbatim by the researchers (SJ and MR), who both participated in all the interviews. The interviews lasted 30-45 minutes.

In order to fulfill the inductive part of the study an open question was posed to the women: “How do you experience the breastfeeding support you have received from your pediatric nurse?” To put the women at ease and encourage them to share their experiences freely, the opening question was followed by such questions as “Please tell us about your breastfeeding experience”, “Please tell me more about.  .   .,”  “How do you mean  .  .  .  ?”, and “Describe your feelings/thoughts when.   .  .”

### 2.4. The MoPPS Scale

In order to fulfill the deductive part of the study and after the open question interviews were conducted, the participants completed the MoPPS scale, either in writing or orally. Both the scale and their responses were later emailed to the participants, and they were asked to explain the motivation behind their answers to each item in a short comment. Their explanations were then returned to the researchers. In this way, the participants got a second chance to reflect on their answers and change the answers if something had been misunderstood during the interview.

The development of the MoPPS scale is part of a larger research project. It is intended to measure experiences of professional support, such as to reflect statements about the experience of informative support given by professionals. Several studies have previously been conducted in order to evaluate and generate understanding about how the scale items are experienced, although further evaluations have been suggested to be necessary [22].

The MoPPS scale is a seven-point Likert scale. Some of the questions are reversed so as to avoid routine answers [23]. The questions on the scale are concerned with how first-time mothers experience breastfeeding support provided by pediatric nurses. The scale contains eight statements: not at all perceptive/perceptive; understanding/not at all understanding; supportive/not at all supportive; had plenty of time/had very little time; provided sufficient information about breastfeeding/did not provide sufficient information about breastfeeding; were calm/were stressed; provided good preparation for the role of parent/provided no preparation for the role of parent; and provided good information about the baby's needs/provided no information about the baby's needs. The statements were divided into negative and positive marks. For example, a negative mark for the statement “not at all perceptive/perceptive” would be awarded if the participant answered between one and three, while a positive mark would be awarded if the participant answered between five and seven. When answering number four it was considered neither positive nor negative.

*Data Analysis*. The transcribed data from* the open question interviews *were analyzed by means of an inductive qualitative content analysis [22]. The text was first read through several times by two of the researchers in order to obtain an overview of the data. It was then divided into meaning units, that is, sentences that share a central meaning. Next, the meaning units where shortened while still preserving the core, which is a process known as condensation [22]. The condensed meaning units were then labelled with a code. Keeping the aim of the study in mind at all times, the researchers read and matched the coded meaning units several times and finally sorted them into different categories and subcategories. In the final step findings were reflected upon in the research team taking the research question into account, and an overall theme emerged, which is considered to be the underlying meaning of the text [22]. Examples of this process are given in Table 1. The analysis of the answers to the MoPPS scale items was performed using a deductive qualitative content analysis [22]. The answers for each item were read through and their meaning was identified. Both positive and negative answers were described (Table 3). In this study, the nurses who work in child health-care centers will be described as pediatric nurses, regardless of what education they have completed. They will also be referred to as “she” or “her,” since all the mothers in this study had female pediatric nurses.

## 3. Results

### 3.1. The Inductive Results

A theme emerged from the results, namely: “When wanting to breastfeed mothers have a desire to be offered professional breastfeeding support”.

The researchers also identified two main categories:* (1) Mothers wanted but lacked breastfeeding support, *and* (2) Mothers received professional support. *Further, a number of subcategories were identified: (1) breastfeeding was experienced as difficult in the beginning, (2) mothers solving problems by themselves when lacking support, (3) mothers did not trust the advices given by the pediatric nurses, (4) distrusting professionals advice due to their lack of interest in breastfeeding, (5) being confirmed and listened to, (6) professionals having knowledge about breastfeeding, and (7) being referred for more breastfeeding support (Table 2).

### 3.2. Mothers Wanted but Lacked Breastfeeding Support

All the mothers in the study wanted to breastfeed their babies and expressed the matter to be important to them. They had to struggle to make breastfeeding work, and most of them experienced some complications during their first attempt at breastfeeding. When the mothers felt a lack of professional support, they felt the need to search for support elsewhere, whether be it a specialized breastfeeding clinic, social support, or information on the internet. When meeting different pediatric nurses and receiving contradictory and unhelpful advice, or when it seemed they were not being listened to, the mothers felt like the breastfeeding support provided to them was not sufficient. The mothers expressed their experience of pediatric nurses as appearing to lack interest in breastfeeding and commented that the nurses tended to focus on bottle feeding rather than breastfeeding support.

*Breastfeeding Was Experienced as Difficult in the Beginning*. The mothers expressed a desire to breastfeed their babies and described how breastfeeding had become important to them. They expressed many feelings regarding breastfeeding, and they reported feeling stressed when it did not work and being afraid of not being able to continue. Breastfeeding was experienced as difficult in the beginning, and the mothers had to struggle to make it work. As first-time mothers, they felt unsure how breastfeeding was supposed to work and what they should do when complications occurred.

The problems that the mothers reported during the first few months of breastfeeding included milk jam, wounds, pain, difficulty latching, and the baby not gaining weight. It was hard to identify a breastfeeding technique that worked. They also experienced trouble reading the baby's signals. As one mother put it:* “I thought it was a rather long learning phase…”*.

“I wasn't ready to give up breastfeeding. Well, of course, in the end if it was the only option... of course, but not if it was possible to do something about it.” (Mother no. 5)

*Mothers Solving Problems by Themselves When Lacking Support*. When the mothers felt that the support they received from the pediatric nurse was not sufficient, they tried to find help elsewhere. Many of them turned to, and received support from, specialized breastfeeding clinics as they thought it was important to receive help from an expert.

Social support also seemed to play an important role in the continuation of breastfeeding. Further, the mothers turned to the internet for information and support regarding breastfeeding. When they did not receive sufficient support in relation to finding products to help them continue breastfeeding, such as a breast pump or nipple shield, they searched for such products and used them on their own initiative.

“…it was more like, go home and see if you can figure it out by yourself.” (Mother no. 7)

*Mothers Did Not Trust the Advice Given by the Pediatric Nurses*. When the mothers felt that they lacked professional support, they often talked about meeting different pediatric nurses and receiving contradictory or unhelpful advices. They felt that the health-care environment did not encourage them to ask about breastfeeding, so they stopped asking for help. Some of them did not trust the advice given by the pediatric nurses. Some were even afraid that their breastfeeding process would be hampered if they followed the pediatric nurse's advice:

*“I really felt that the advice the pediatric nurse gave might make me produce less and less milk, and it really made me worried, because I really wanted to continue breastfeeding.”* (Mother no. 5)

Sometimes, the mothers perceived the nurses' focus to be on other things than making breastfeeding work, for example, the baby gaining weight. They also felt that they were not being listened to and that their opinions were not respected. Many of them felt that they did not receive the support they needed, or perhaps even any support at all, even though they asked for it. They felt that the pediatric nurses decided and told them what to do rather than discussing things and involving them in decisions.

“It was hard knowing that I didn't get the support I wanted.” (Mother no. 2)

*Distrusting Professional's Advice due to Their Lack of Interest in Breastfeeding*. The mothers were surprised about how little focus the pediatric nurses placed on breastfeeding. Seemingly, they neither talked, nor asked the mothers, about breastfeeding. The pediatric nurses were mostly focused on the baby gaining weight, and not on how the baby was being fed. When breastfeeding proved insufficient to feed the baby, the mothers felt that the focus was on introducing formula by bottle rather than helping them to establish effective breastfeeding. They also felt that the pediatric nurses exhibited low interest and knowledge concerning breastfeeding and that it was not really part of their profession.

When asked about what kind of support they desired in terms of breastfeeding, the mothers wanted more interest, information, advice, tools, and support in general from the pediatric nurses. The mothers wished for breastfeeding observation, and they wanted the pediatric nurses to show them different positions and help the baby to latch properly. The mothers wanted concrete facts and clear answers to their questions. In order to protect their breastfeeding practice, the mothers wanted the pediatric nurses to offer them solutions other than formula feeding by bottle. It was considered important to be heard and listened to, and the mothers wanted the pediatric nurses to acknowledge their difficulties and show interest in breastfeeding. A good relationship and the ability to have a discussion were also desired by the mothers. The establishment of a connection between care chains (such as having easy access to a specialized breastfeeding clinic), or a referral to a specialist if their questions and problems were beyond the pediatric nurses' knowledge, were also desired by the mothers. Some of them also asked for more breastfeeding information prior to labor.

“.. like offering suggestions about different breastfeeding positions that I could try…” (Mother no. 9)

### 3.3. Mothers Received Professional Support

If mothers were to experience good breastfeeding support, it was considered important that they were listened to and their difficulties acknowledged. It was also considered important that their partners were involved in the breastfeeding process and that extra appointments were available if needed. The mothers felt that they received good support if the pediatric nurses exhibited a good level of knowledge regarding breastfeeding and if they referred the mothers for specialist help when needed.

*Being Confirmed and Listened to*. When talking about the support they had received, the mothers mentioned that they had been heard and listened to. The pediatric nurses had asked them questions and reassured them that they were doing the right thing. The nurses had been supportive, which included providing the right kind of support, and they had a good, honest relationship with the mothers. When the whole family was involved in the breastfeeding process, the mothers felt they were not alone.

An important aspect of being seen and acknowledged by the nurses concerned the option of having extra appointments when the mothers felt insecure. They also experienced good support when the pediatric nurses did not push them to start bottle feeding against their will. They preferred it when the nurses encouraged them to continue breastfeeding, had no preconceived ideas when they first met, listened to the mothers' wishes, and supported the mothers' decisions.

“When we met, the pediatric nurse always talked to us both. And not just to me.” (Mother no. 3)

*Professionals Having Knowledge about Breastfeeding*. When the pediatric nurses appeared to be up-to-date and knowledgeable regarding breastfeeding, the mothers felt like they received good support. The mothers received help in relation to identifying better breastfeeding positions when they experienced problems, and the nurses searched for information when they did not know the answers.

“She has been really good. She has found more information… tried to find more information to be able to help me and that has been nice.” (Mother no. 1)

*Being Referred for More Breastfeeding Support*. If the mothers were referred to other professionals for breastfeeding support when the nurses felt they were not able to provide them with the requested support, the mothers felt satisfied with the level of support on offer. Yet, when the pediatric nurses referred the mothers to specialists without even attempting to help them with their breastfeeding problems, the mothers felt a lack of professional support. A referral could be made to a colleague, a dietician, or a specialized breastfeeding clinic where the mothers could get more help with their problems. It was considered valuable when the pediatric nurses helped them to get in contact with specialists.

“…she referred me to the specialized breastfeeding clinic instead.” (Mother no. 4)

### 3.4. Summarizing the Inductive Results

The results showed that while the mothers all wanted to breastfeed their babies, they all experienced different problems, which made them seek professional support, although not all of them received the level of support they desired. They wanted the pediatric nurses to recognize their individual needs and support them in their desire to breastfeed. When they felt listened to, supported, and acknowledged, the mothers believed that they had control and were in charge of their breastfeeding process. When the pediatric nurses focused on things other than breastfeeding, the mothers did not feel like the health-care environment encouraged them to talk about breastfeeding. Some of the mothers also doubted the pediatric nurses' level of interest and knowledge concerning breastfeeding, and therefore they searched for breastfeeding support elsewhere.

### 3.5. The Deductive Results

An overview of the results of the MoPPS scale is shown in Table 3. An explanation of the meaning of each item is followed by the answers given by the mothers, which are divided into negative and positive assessments. For one item, one of the mothers responded with a positive comment, but answered with a negative mark.

### 3.6. Comparing the Inductive and Deductive Results

The results concerning the inductive and deductive results will be compared as follows. The mothers who were unsatisfied with the support they received described the pediatric nurses as not listening, not being supportive, and not understanding their wish to breastfeed. This was also seen in relation to the MoPPS scale, both in terms of the answers given to the scale and the explanations offered by the mothers regarding their answers. The mothers who had experienced good breastfeeding support provided examples of the pediatric nurses being supportive of their needs, perceptive, and offering extra appointments when needed. The mothers talked about how the pediatric nurse involved her partner in the breastfeeding process, although the MoPPS scale does not contain a question about the involvement of partners.

The MoPPS scale contains questions that describe what is important when providing breastfeeding support, for example, time, providing information, being supportive, being understanding, and being perceptive as to the mother's wish to breastfeed. It also separates breastfeeding support from support regarding parenting and information about the baby, which the mothers described in the interviews as being separate concepts. However, it was found that the mothers considered it important that the pediatric nurses exhibited sufficient knowledge about breastfeeding. It was also seen as important that the pediatric nurses involved the partner in the breastfeeding support. Therefore, we suggest that these aspects should be included in the MoPPS scale for pediatric nurses.

## 4. Discussion

The findings of this study showed agreement between the mothers' answers to the open questions and their comments on the MoPPS scale. By using semistructured qualitative interviews, the researchers were able to explore the mothers' experiences. The mothers expressed a need for professional breastfeeding support. They also acknowledged that it was difficult being a first-time mother and not knowing how breastfeeding was supposed to work. They wanted the pediatric nurses to be the ones who provided them with breastfeeding support, since they were the health professionals that the mothers met most often. In contrast to their responses to the MoPPS scale, the mothers expressed that the pediatric nurses should be more understanding of their need for professional support. The pediatric nurses should perhaps put extra effort in supporting first-time mothers in relation to their breastfeeding situation, since they do not have anything to relate to. Feelings of uncertainty regarding breastfeeding, not knowing what to expect, and not knowing if it will work were common [7, 25]. In the beginning, breastfeeding mothers may experience a so-called reality check, which will likely be accompanied by feelings of frustration and disappointment, since their breastfeeding experience did not turn out as they expected. It is commonly expected that breastfeeding is easy, natural, and something that simply works [26].

The results of the present study showed that the mothers expressed a great desire to breastfeed their babies, and they were sad when they were not able to do so. They talked about how hard it could be, as well as the many difficulties associated with breastfeeding that could occur. The importance of pediatric nurses being supportive and identifying the mothers' individualized needs was made clear in the MoPPS scale. Other studies show that mothers find it difficult to establish breastfeeding, struggle to make the baby latch, and experience physical complications such as sore nipples, pain, and milk jams [5, 7, 25, 26]. This could indicate that breastfeeding awakens many feelings in the mothers and also that they are willing to undergo a difficult experience in order to make breastfeeding work. This might render them vulnerable to different opinions and inadequate treatment. Some prior studies reported that breastfeeding complications, lack of knowledge, and poor breastfeeding support were the key reasons why women ceased breastfeeding [5, 6]. Professionals' perceived lack of interest in breastfeeding was expressed by the mothers in this study. This made them feel that child health-care centers were not the best place to ask for breastfeeding support. They also felt that there was a lack of focus on breastfeeding. These results are in line with other research showing that giving formula by bottle was seen as the normal way of feeding a baby, while breastfeeding was seen as almost abnormal behavior [27].

The credibility of the support provided has been found to be strengthened when professionals create an open environment and recognize the individualized needs of the patient [16]. Health-care professionals and mothers might have different views about breastfeeding support.

Interestingly, health-care professionals experienced themselves as being very supportive, whereas the mothers experienced the support provided as being insufficient and superficial [17]. In another study, interviewing both mothers and health-care professionals, health-care professionals' attitudes toward breastfeeding are shown to be of importance in order to provide support to mothers. Health-care professionals are worried about providing “too much breastfeeding support”, arguing that they had to appear “nonbiased” during their contact with patients. Breastfeeding is also seen as something that is good for mothers to try, although the professionals understand if the mothers do not want to try it. Additionally, if the mothers need extensive support, it is considered better that they went to lactation consultants [28].

The results from our study also showed that professional breastfeeding support offered from pediatric nurses was much too often experienced by the mothers to be poor, unhelpful with contradictory advice. This could be related to the fact that the mothers meet different pediatric nurses at the pediatric health-care centers. It can also be related to the mothers being offered professional breastfeeding support from specialized breastfeeding clinics and that this support was in conflict with the support from the pediatric nurses. This result is in line with the results of another study, which found that conflicting advice was common when the relationship between health-care staff and mothers was insufficient or when there was a lack of continuity [29]. Results in this study also showed that when mothers experience that professional breastfeeding support is conflicting or not offered in relation to their unique needs, especially when complications occur, mothers actually experience more complications or even cease breastfeeding. The feeling of being alone in this situation, as well as not being seen and reassured, could leave mothers so unsure about their own knowledge that they begin to doubt themselves and their ability to breastfeed.

In contrast the results showed that mothers felt they were supported and could ask for help, when the nurses exhibited good knowledge regarding breastfeeding. They hence might not feel that they need to search for advice elsewhere. Since the mothers in this study felt positive about being referred to a specialist when the pediatric nurses did not know how to help them, it was considered important that the nurses were aware of their abilities and shortcomings. A good relationship between the care giver and the mother has been recognized as important, since it often results in more individualized care [26]. In order to provide adequate breastfeeding support, it is important that health-care staff listen to and acknowledge each individual's specific needs [17]. Having enough time was seen by the mothers as crucial in relation to receiving good support. Health-care professionals indicate that breastfeeding mothers are in need of additional support and that they want to provide such support, although they complained about their lack of time [27]. Mothers in this study talked about the benefits of having a good relationship with their pediatric nurses, as well as how they felt supported and reassured with regard to their choices. Parallels can be drawn with the results of the MoPPS scale, which indicated that nurses being perceptive of mothers' thoughts and supportive of their decisions were vital. Being heard might allow the mothers to feel that they are in ultimate charge of their breastfeeding, and it might help them to determine what they should do if it did not work. Their self-confidence might increase, which might in turn inspire them to continue breastfeeding.

### 4.1. The Mothers Described the Questions in the MoPPs Scale as Easy to Understand

They also commented that it was good to have many response options ranging from positive to negative, as well as to be able to answer in the middle. They were positive with regard to the questions about preparation for the parenting role and information about the needs of the baby, since they saw such matters as separate from the issue of breastfeeding support. They experienced the pediatric nurses to be good at dealing with these matters, even if they experienced the breastfeeding support as bad. One of the mothers wanted all the positive answers to be on one side, while one of them thought it was good that the answers were mixed as that meant you had to think about how you answered. For one of the scale items, one mother answered with a negative mark, but commented with a positive answer. This could render the results of the scale untrustworthy if the mothers did not pay attention to the positive and negative sides for each question. Yet, this type of questionnaire can cause the mothers to really think about their answers and not simply put all the marks on one side of the scale. It is possible that there could be a clearer difference between the positive and negative sides of the scale, perhaps marked with a different color or with “not” underlined, so that respondents could more easily see which side is positive for each question. In a prior study concerning the MoPPS scale, the authors found that a question about knowledge/competence could improve the scale, since the mothers described this issue to be important [21]. One mother experienced good support when the pediatric nurse involved her partner in the breastfeeding support, which is in line with earlier research that partner support is important for breastfeeding success [30]. This could represent another item to be added to the MoPPS scale, since social support can be important in relation to the continuation of breastfeeding. The mothers in this study found the questions in the scale to be easy to understand. Similar results have been found in other studies, which found that the scale was both relevant and simple to understand [20, 31].

### 4.2. Limitations and Strengths of the Study

The limitation of this study is the small sample size. More participants could give a different result but the variation among participants and richness of their narratives were considered to enhance the trustworthiness of the study [32]. When using convenience sampling, the pool of participants might be limited, since only those mothers who were interested in talking about their breastfeeding situation were likely to offer to participate. The participants could hence be those who received good support or those who experienced a lack of professional support. However mothers in this study provided a variation of experiences which was considered a strength for trustworthiness of the study [32]. A different means of recruiting participants might result in the identification of broader experiences of professional breastfeeding support. However, the fact that the results were derived from both the deductive and inductive parts of the study could be considered a strength of the research. Since some of the interviews were conducted by phone, there is always a risk of missing out on information since the researchers are not able to read the expressions of body language from the participants. On the other hand, using the telephone may increase the level of comfort for the participant, which could result in a more relaxed interview [33]. When using a qualitative design, the researchers will always be cocreators of the results [22]. In order to prompt the participants to share their own experiences, an open question was used for the inductive part of the study. Two of the researchers (MR, SJ) participated in all the interviews, as well as in all analyzing of the data, which allowed them to reflect together on the results. This could also increase the trustworthiness of the study. Transferability of findings from qualitative studies need a detailed description of participants and environment for the study [32], which have been taken into consideration in this study.

## 5. Conclusion

The results of this study highlighted the importance of pediatric nurses having up-to-date knowledge regarding breastfeeding in order to be able to offer professional breastfeeding support to mothers. It is also important to offer individualized support to mothers, as well as listen to their wishes concerning breastfeeding. Such knowledge could be used by any professionals tasked with providing breastfeeding support, including neonatal care nurses and midwifes working on maternity wards.

The MoPPS scale can be a useful tool for helping pediatric nurses to offer mothers professional breastfeeding support. Indeed, when offering breastfeeding support, pediatric nurses can use the items included on the MoPPS scale as guidance. A clearer indication of which side is positive and which is negative would be useful in terms of helping respondents to avoid answering with an incorrect mark. An addition in the MoPPs scale could be questions about the professionals' knowledge/competence and about the partners' involvement in breastfeeding support, since social support is known to assist with the initiation and continuance of breastfeeding.

## Figures and Tables

**Table 1:** Examples of condensation, code, subcategory, and category.

Condensation	Code	Subcategory	Category
Felt stressed when breastfeeding did not seem to work	Stress when breastfeeding did not work	Breastfeeding was experienced as difficult in the beginning	Mothers wanted but lacked breastfeeding support
It was hard to read the baby's signal in the beginning	Difficulties reading the baby's signals		
I turned to a specialized breastfeeding clinic	Turned to a specialized breastfeeding clinic	Mothers solving problems by themselves when lacking support	
The advice the pediatric nurse gave do not work for me	Unhelpful advice	Mothers did not trust the advices given by the pediatric nurses	
The focus was on making sure the baby got food and the easiest way was formula	Focus on formula feeding rather than breastfeeding	Distrusting professionals advice due to their lack of interest in breastfeeding	

Confirmed my need for support	Confirmation	Being confirmed and getting listened to	Mothers received professional support
The pediatric nurse updated her knowledge to be able to provide more support	Updated knowledge to provide support	Professionals having knowledge about breastfeeding	
The pediatric nurse helped me to obtain more specialized help	Referred to a specialized breastfeeding clinic	Being referred for more breastfeeding support	

**Table 2:** Overview of theme, categories, and subcategories.

Theme	Categories	Subcategories
When wanting to breastfeed, mothers have a desire to be offered professional breastfeeding support	Mothers wanted but lacked breastfeeding support	Breastfeeding was experienced as difficult in the beginning
		Mothers solving problems by themselves when lacking support
		Mothers did not trust the advices given by the pediatric nurses
		Distrusting professionals advices due to their lack of interest in breastfeeding
	Mothers received professional support	Being confirmed and listened to
		Professionals having knowledge about breastfeeding
		Being referred for more breastfeeding support

**Table 3:** Assessment of the MoPPS scale.

MoPPS scale item	Answers: lowest to highest (n=9)	Overall meaning of the scale item	Meaning when responding with a negative mark	Meaning when responding with a positive mark
Not at all perceptive (1) Perceptive (7)	2–7	Being perceptive about the mother's thoughts and supportive of her decisions	Nonresponsive; no discussion when asked; not helping with the real problem; not listening	Responsive to my thoughts; supportive of my decisions; responsive; empathetic; interested

Not at all understanding (1) Understanding (7)	2–7	Understanding of the mother's need for professional support	Not understanding even though being told the mother wanted to breastfeed	Understanding breastfeeding was important; understanding the problems being experienced

Supportive (1) Not at all supportive (7)	1–6	Identified the mother's individualized need; provided the support she wanted	Inadequate breastfeeding support; not listening to the desire for exclusive breastfeeding; simply referred to specialist; did not provide the help that was wanted	Booking extra appointments; supportive of choice; listening and understanding

Had very little time (1) Had plenty of time (7)	2–7	Gave the time the mother wished for	Seemed stressed sometimes; visits felt stressful; only focused on the weight of the baby	Had plenty of time for mother and baby; always had time to talk about what felt important; lots of time; easy to get an appointment; offered extra appointments when wanted; lots of time, but did not get a home visit

Provided sufficient information about breastfeeding (1) Did not provide sufficient information about breastfeeding (7)	1–7	Pediatric nurse able to provide the support required	Did not receive any breastfeeding information; were referred; not enough knowledge	Searched for more knowledge to be able to provide support; good information when pregnant; had the time needed

Being calm (1) Being under stress (7)	1–3	Pediatric nurse calm during meeting	Not relevant	Seemed calm while focusing on the baby; never experienced any stress; felt calm; appreciated her being calm; calm even when pressed for time; lack of breastfeeding support was not due to being stressed

Provided good preparation for the role of parent (1) Provided no preparation for the role of parent (7)	1–5	Pediatric nurse talked about the parenting role	Just focused on the baby; did not seem engaged in supporting the parenting role	Talked a lot about the parenting role and the differences that could occur; understanding towards both parents; asking questions; was personal; willing to talk about parenthood; offered support

Provided no information about the baby's needs (1)	4–7	Pediatric nurse had good knowledge and focused on the baby	Not relevant	Support with how the baby was doing; listening and providing advice; repeated information; good

Provided good information about the baby's needs (7)				Knowledge; wanted more information about how to get on with daily life; focusing on the baby

## Data Availability

The data used to support the findings of this study are available from the corresponding author upon request. Our interview data are recorded and the recordings are safely kept for at least 10 years after publication.

## Abbreviations

MoPPS scale: Mother Perceived Support from Professionals scale
WHO: World Health Organization
BFHI: Baby-Friendly Hospitals Initiative
UNICEF: United Nations Children's Fund.
